# Qishen granules regulate intestinal microecology to improve cardiac function in rats with heart failure

**DOI:** 10.3389/fmicb.2023.1202768

**Published:** 2023-06-15

**Authors:** Kuo Gao, Xue Yu, Fanghe Li, Yiran Huang, Jiali Liu, Siqi Liu, Linghui Lu, Ran Yang, Chao Wang

**Affiliations:** ^1^School of Chinese Materia Medica, Beijing University of Chinese Medicine, Beijing, China; ^2^Guanganmen Hospital, China Academy of Chinese Medical Sciences, Beijing, China; ^3^Zang-xiang Teaching and Research Department, The Institute of Basic Theory for Chinese Medicine, China Academy of Chinese Medical Sciences, Beijing, China

**Keywords:** Qishen granule (QSG), heart failure, cardiac functions, intestinal microecology, traditional Chinese herbs

## Abstract

**Introduction:**

Qishen Granule (QSG), a clinically approved traditional Chinese medicine, has been researched for treating heart failure (HF) for many years. However, the effect of QSG on intestinal microecology remains unconfirmed. Therefore, this study aimed to elucidate the possible mechanism of QSG regulating HF in rats based on intestinal microecological changes.

**Methods:**

A rat model with HF induced by myocardial infarction was prepared by left coronary artery ligation. Cardiac functions were assessed by echocardiography, pathological changes in the heart and ileum by hematoxylin–eosin (HE) and Masson staining, mitochondrial ultrastructure by transmission electron microscope, and gut microbiota by 16S rRNA sequencing.

**Results:**

QSG administration improved cardiac function, tightened cardiomyocytes alignment, decreased fibrous tissue and collagen deposition, and reduced inflammatory cell infiltration. Electron microscopic observation of mitochondria revealed that QSG could arrange mitochondria neatly, reduce swelling, and improve the structural integrity of the crest. Firmicutes were the dominant component in the model group, and QSG could significantly increase the abundance of Bacteroidetes and Prevotellaceae_NK3B31_group. Furthermore, QSG significantly reduced plasma lipopolysaccharide (LPS), improved intestinal structure, and recovered barrier protection function in rats with HF.

**Conclusion:**

These results demonstrated that QSG was able to improve cardiac function by regulating intestinal microecology in rats with HF, suggesting promising therapeutic targets for HF.

## 1. Introduction

Heart failure (HF) can arise from structural or functional abnormalities of the heart due to various reasons and is the final stage of multiple cardiovascular diseases (Mcmurray et al., 2012).

With improvements in global economic levels, changes in human dietary structures, and poor lifestyle habits, HF has become a worldwide public health issue. A systematic review by Askoxylakis et al. revealed that the 5-year mortality rate of chronic heart failure is essentially equal to that of cancer (Askoxylakis et al., 2010). Studies indicate that there are approximately 26 million heart failure patients globally, with a prevalence of 1.5% to 2.0% in developed countries, and over 10% among individuals aged 70 and above (Mosterd and Hoes, 2007).

With the development of modern medicine, researchers have deepened their understanding of HF, leading to changes in concepts, innovations in methods, and updates in guidelines. While the in-hospital fatality rate of HF patients has shown a clear downward trend, it is worth noting that the re-hospitalization rate continues to increase (Hu, 2021). Therefore, it is still a hot and difficult topic in clinical research to improve the quality of life, reduce the fatality and re-hospitalization rate, and thus promote the long-term prognosis (Mao et al., 2021).

The intestine, also known as the second brain, is the body's largest digestive and excretory organ (Mayer, 2011). The intestinal microbiota primarily consists of Firmicutes, Bacteroidetes, Proteobacteria, Actinobacteria, and Verrucomicrobia (Eckburg et al., 2005), with over 90% of bacteria in a healthy gut classified as Bacteroidetes and Firmicutes (Gill et al., 2006). Under normal physiological conditions, the host provides a suitable environment and necessary nutrition for gut microbiota, which in turn participate in various biological functions, such as nutrient metabolism and absorption, energy balance, neural development, immune regulation, and maintenance of intestinal mucosal barrier defense (Everard and Cani, 2014). This creates a balanced, symbiotic, and ecological environment, leading to the gut microbiota gene being referred to as the second genome (Mayer, 2011). In an unbalanced state, gut microbiota dysbiosis can impact the host's growth, development, health and disease, and drug treatment (Hooper and Gordon, 2015). The disorder is closely related to the onset and progression of various diseases, including those in the digestive system (Larsson et al., 2012), mental system (Wang B. et al., 2017), endocrine system (Bäckhed et al., 2004; Qin et al., 2012; Tremaroli and Bäckhed, 2012), and autoimmune system (Tremaroli and Bäckhed, 2012), as well as some infectious diseases (Wang et al., 2014). Gut microbiota dysbiosis and its metabolites play a vital role in the occurrence and development of HF (Sandek et al., 2007a; Andreas et al., 2014). Therefore, interventions targeting gut microbiota dysbiosis, improving intestinal membrane barrier function and intestinal wall permeability, and reducing endotoxin absorption and inflammation may alleviate myocardial damage, suggesting a new direction for HF treatment in the future.

Qishen granule (QSG), a clinically approved traditional Chinese medicine, has been researched for treating HF for many years (Wang J. et al., 2017). QSG is composed of six botanical drugs, including *Astragalus camptoceras* Bunge (*Fabaceae), Aconitum carmichaelii* Debeaux (*Ranunculaceae*), *Salvia miltiorrhiza* Bunge (*Lamiaceae*), *Scrophularia ningpoensis* Hemsl. (*Scrophulariaceae*), *Lonicera japonica* Thunb. (*Caprifoliaceae*), and *Glycyrrhiza uralensis* Fisch. ex DC. (*Fabaceae*) (30: 9: 15: 10: 10: 6) (Chen et al., 2022; Li et al., 2022). Our previous study detailed its preparation process and composition identification (Wang et al., 2012; Xia et al., 2017).

However, the effect of QSG on intestinal microecology remains unconfirmed. Therefore, this study aimed to elucidate the potential mechanism of QSG regulating HF in rats, based on intestinal microecological changes.

## 2. Materials and methods

### 2.1. Experimental animals and Ethics Statement

Male Sprague–Dawley rats (180 ± 10 g) were provided by the Vital River Laboratory Animal Technology Co. Ltd. (Beijing, China). The animal housing conditions were maintained at 23 ± 2°C, 40 ± 5% relative humidity, and 12:12 h light–dark cycles. Rats were adaptively fed for 1 week. All experimental procedures were conducted and supervised by the Animal Care Committee of Beijing University of Chinese Medicine, in accordance with the National Institute of Health Guide for the Care and Use of Laboratory Animals.

### 2.2. Drugs

Qishen Granule is composed of six botanical drugs, including *Astragalus camptoceras* Bunge (*Fabaceae), Aconitum carmichaelii* Debeaux (*Ranunculaceae*), *Salvia miltiorrhiza* Bunge (*Lamiaceae*), *Scrophularia ningpoensis* Hemsl. (*Scrophulariaceae*), *Lonicera japonica* Thunb. (*Caprifoliaceae*), and *Glycyrrhiza uralensis* Fisch. ex DC. (*Fabaceae*) (30: 9: 15: 10: 10: 6) (Chen et al., 2022; Li et al., 2022), and its composition was identified by high-performance liquid chromatography (Wang et al., 2012; Xia et al., 2017).

Trimetazidine, used as the positive drug, was purchased from Servier (Tianjin) Pharmaceutical (National drug approval number H20055465).

### 2.3. HF model induction and Electrocardiogram

As previously described (Gao et al., 2020), ligation surgery of the left anterior descending (LAD) coronary artery was performed on anesthetized rats using intraperitoneal injection of 1% pentobarbital sodium (45 mg/kg). Briefly, a left thoracotomy was performed between the third and fourth intercostal spaces in the rats. After exposing the cardiac tissues, the LAD was ligated with a sterile suture (Shuangjian, Shanghai, P. R. China) 1 mm below the left atrium. The thorax was then closed layer by layer. After thoracotomy, rats were warmed on a heated blanket. Sham-operated rats underwent the same procedure without LAD ligation. On the third day after surgery, the rats were anesthetized by intraperitoneal injection of 1% pentobarbital sodium at 40 mg/kg, and the presence of 6–8 pathological Q-waves in the electrocardiogram indicated successful surgical ligation (result shown in Supplementary Figure 1).

### 2.4. Animal grouping and drug administration

Rats with successful HF models were randomly divided into the Model group (Model), Qishen granule group (QSG), and trimetazidine group (TMZ), with 12 animals in each group. As previously described (Gao et al., 2020), rats were treated with intragastric administration at a daily dose of 18.66 g/kg for 28 days. Rats in the TMZ group received 6.3 mg/kg of TMZ. Rats in the sham operation group and model group were administered an equal volume of normal saline intragastrically for 28 days.

### 2.5. Assessment of cardiac functions by echocardiography

As previously described (Gao et al., 2020), M-mode echocardiography was used to measure the internal diameter of the left ventricle at the end of systolic/diastolic periods and the thickness of the anterior/posterior left ventricle. Then left ventricular ejection fraction (LVEF), Z left ventricular fractional shortening (LVFS), left ventricular anterior wall; diastolic (LVAW;d), left ventricular anterior wall; systolic (LVAW;s), left ventricular internal end-diastolic diameter (LVID;d), left ventricular internal end-systolic diameter (LVID;s), left ventricular posterior wall; diastolic (LVPW;d), and left ventricular posterior wall; systolic (LVPW;s) were calculated to evaluate the cardiac systolic function and myocardial hypertrophy.

### 2.6. Hematoxylin–eosin (HE) and Masson staining

The heart and ileum tissues were fixed in 4% paraformaldehyde for more than 48 h, embedded in paraffin, and sectioned at 5 μm thickness for further histological analysis. Hematoxylin–eosin (HE) staining was performed for the heart and ileum tissues, and Masson staining was performed for the heart tissues to visualize tissue architecture.

### 2.7. Mitochondrial ultrastructure observation using transmission electron microscopy

Cardiac tissues from the infarct border zone of the left ventricle (1 mm × 1 mm × 2 mm) were fixed in 4% glutaraldehyde (2 h), in 1% osmic acid (2 h), and then washed with phosphate-buffered saline (PBS) solution three times (5 min). Ultrastructural alterations were observed using a transmission electron microscope (Hitachi, Tokyo, Japan) after dehydration, permeation, embedding, and ultrathin sections were cut.

### 2.8. LPS detection

Lipopolysacchride detection was measured following the instructions of the test kit (20152400090, Fuzhou Xinbei Biochemistry Industry Co., Ltd).

### 2.9. Detection of gut microbiota

Fresh fecal samples (1 g) were collected from rats and placed in a 30 ml sterile tube containing 15 ml of phosphate-buffered saline (pH 7.2). The samples were mixed and centrifuged at 200 RPM for 10 min. After removing the sediment, 200 ul of suspension was obtained after oscillation. DNA extraction, PCR amplification, Illumina MiSeq sequencing, and processing of sequencing data are described in detail in the Supplementary Material. The alpha diversity indexes, including species rarefaction curve, richness index (Sobs index), and diversity index (Shannon index), were analyzed using Mothur (Schloss et al., 2011) (version v.1.30.1), and the similarity level of operational taxonomic units (OTUs) for index evaluation was 97% (0.97). Beta diversity analysis was performed to compare differences among different groups, including partial least squares discriminant analysis (PLS-DA) and linear discriminant analysis effect size (LEfSe).

The raw reads were deposited into the NCBI Sequence Read Archive (SRA) database (Accession Number: SRP431350).

### 2.10. Statistical analysis

Data are presented as the mean ± standard error (X ± SEM). One-way ANOVA or Kruskal–Wallis H analysis of variance were used to detect statistically significant differences (*P* < 0.05) among groups. A community histogram was drawn using GraphPad Prism (version 9.0), and Venn diagrams, heatmaps, and PLS-DA plots were created using R packages.

## 3. Experimental results

### 3.1. QSG improved cardiac functions in HF rats

**Figure 1:**
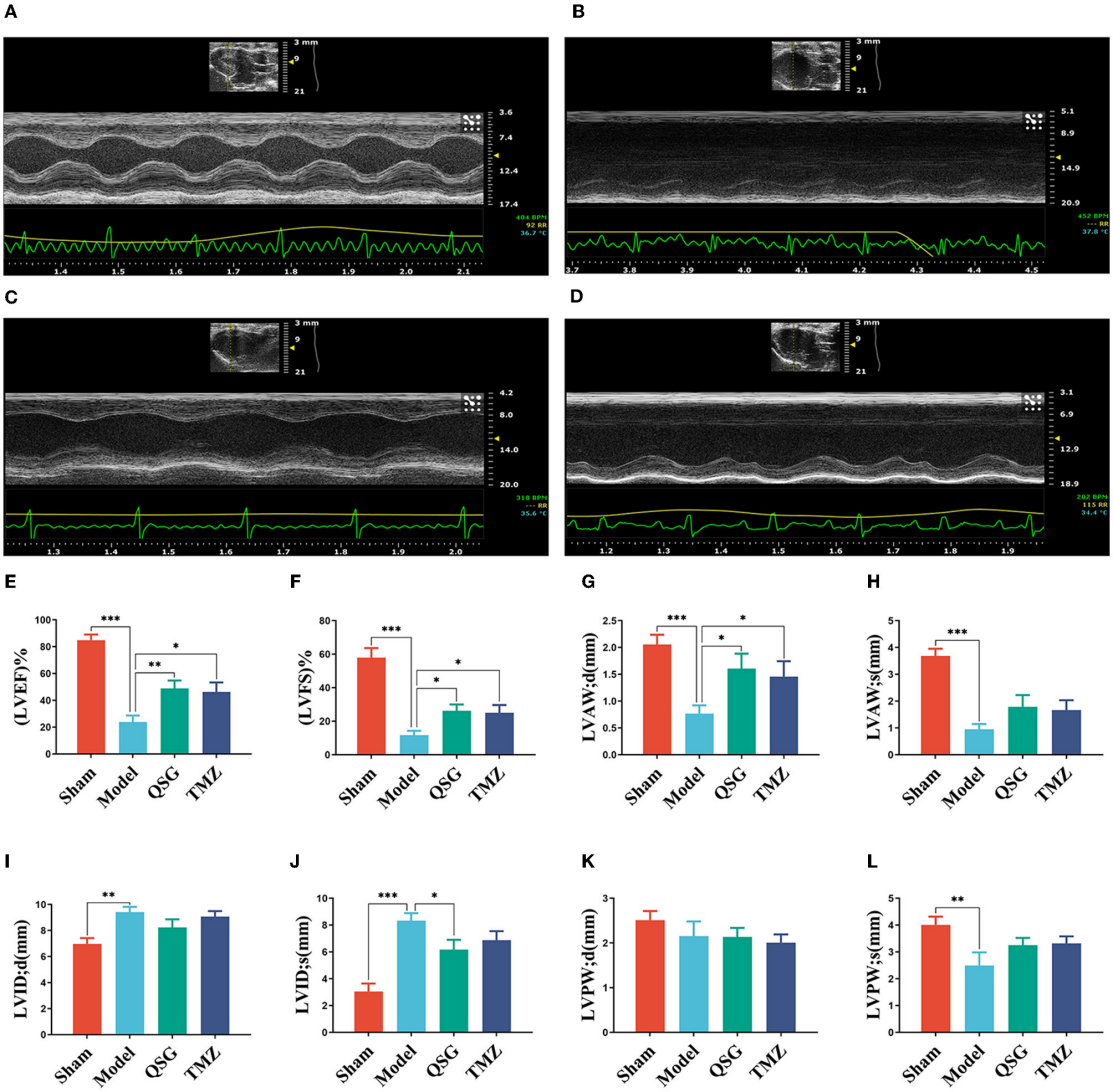
QSG improved cardiac function and reduced pathological changes in HF rats. **(A)** Sham group. **(B)** Model group. **(C)** Qishen granule group. **(D)** TMZ group. **(E–L)** LVEF, LVFS, LVAW;d, LVAW;s, LVID;d, LVID;s, LVPW;d, and LVPW;s *N* = 6 per group. *P < 0.05, **P < 0.01, ***P < 0.001 vs. model group.

Echocardiography results (Figure 1) demonstrated significant downregulation of LVEF, LVFS, LVAW;d, LVAW;s, LVPW;d, and LVPW;s (*P* < 0.001, *P* < 0.01) and upregulation of LVID;d and LVID;s (*P* < 0.001, *P* < 0.01) in the model group compared to the sham group. QSG and TMZ significantly increased LVEF, LVFS, and LVAW;d (*P* < 0.01, *P* < 0.05), while QSG also significantly improved LVID;s (*P* < 0.05).

### 3.2. QSG reduced pathological changes in HF rats

**Figure 2:**
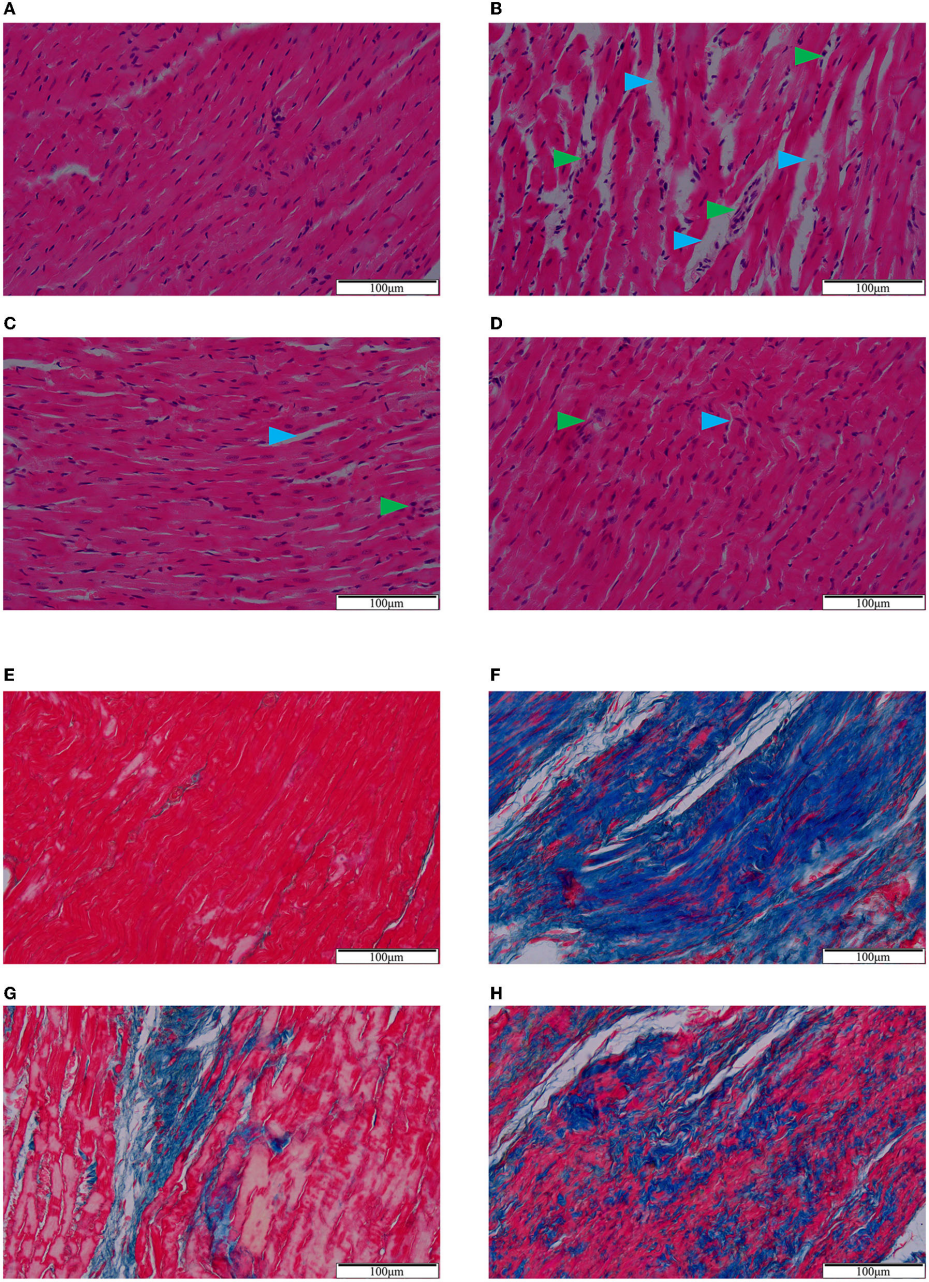
HE **(A–D)** and Masson **(E–H)** staining. **(A/E)** Sham group. **(B/F)** Model group. **(C/G)** Qishen granule group. **(D/H)** Trimetazidine group. Blue arrowheads indicate the intercellular space and green arrowheads indicate inflammatory infiltration **(A–D)**. Blue-stained fiber indicates collagen deposition **(E–H)**. Scale bar = 100 μm.

Based on HE staining (Figures 2A–D), cardiomyocytes in the sham group were tightly arranged and orderly, while those in the model group were loosely arranged with obvious inflammatory cell infiltration and pyknotic dark-staining nuclei. Compared with the model group, QSG and TMZ attenuated these pathological changes.

Masson staining (Figures 2E–H) showed that, compared to the sham group, the cardiomyocytes in the model group were necrotic and replaced by extensive collagen fibrous tissues with diffuse and infiltrating distributions. Both QSG and TMZ significantly inhibited collagen deposition.

### 3.3. QSG improved the structure of mitochondria

**Figure 3:**
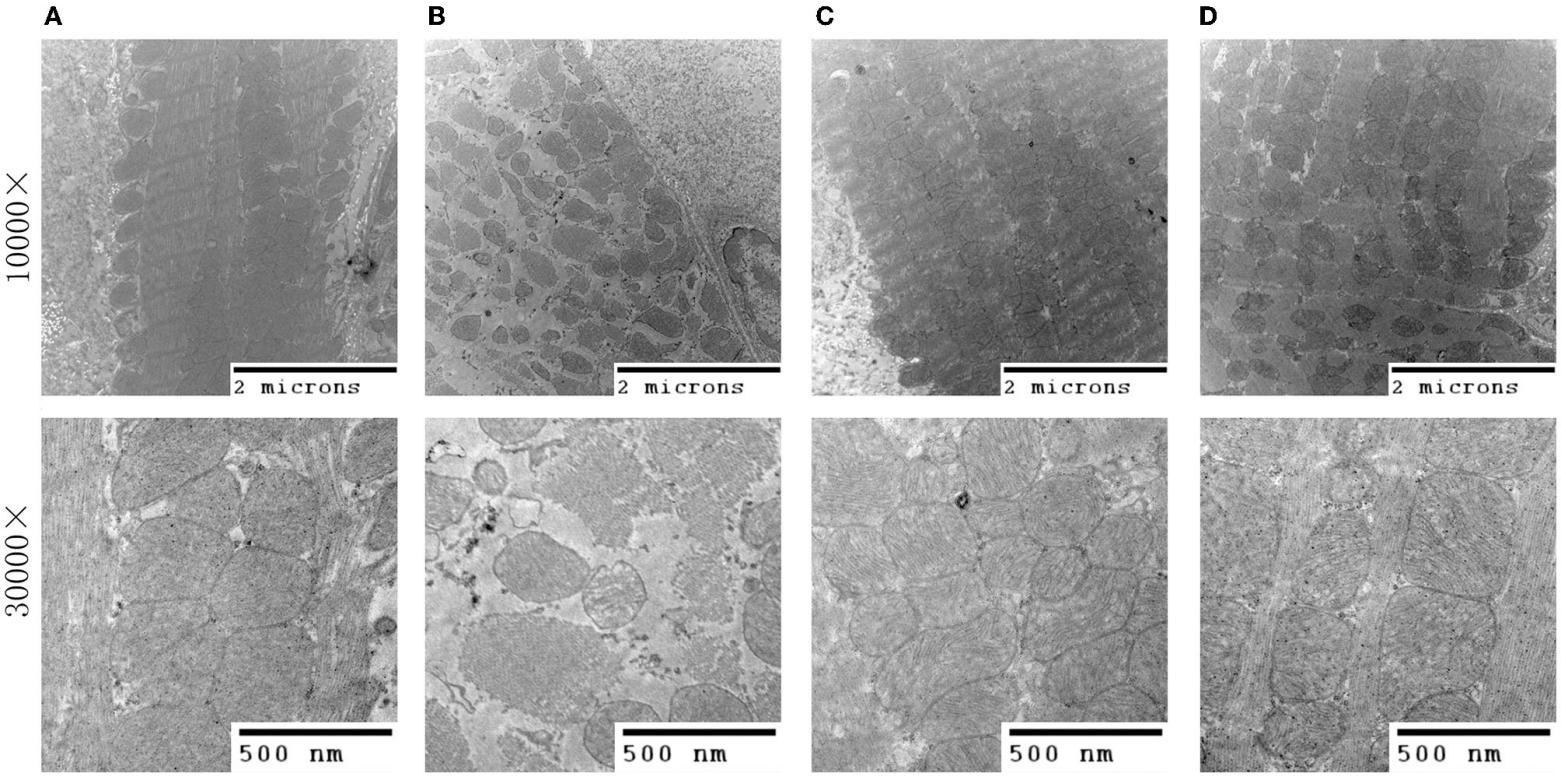
Transmission electron microscopy of myocardial tissue. **(A)** Sham group. **(B)** Model group. **(C)** Qishen granule group. **(D)** Trimetazidine group.

Transmission electron microscopy results (Figure 3) revealed that in the sham group, mitochondria with a complete membrane structure were round or oval and densely and orderly arranged. The mitochondrial cristae and matrix were arranged evenly and clearly (Figure 3A). In contrast, the model group exhibited scattered arrangement, obvious swelling, a loose matrix, and evident partial fracture cristae (Figure 3B). QSG and TMZ significantly improved the structure of mitochondria.

### 3.4. QSG reduced pathological changes in the ileum

**Figure 4:**
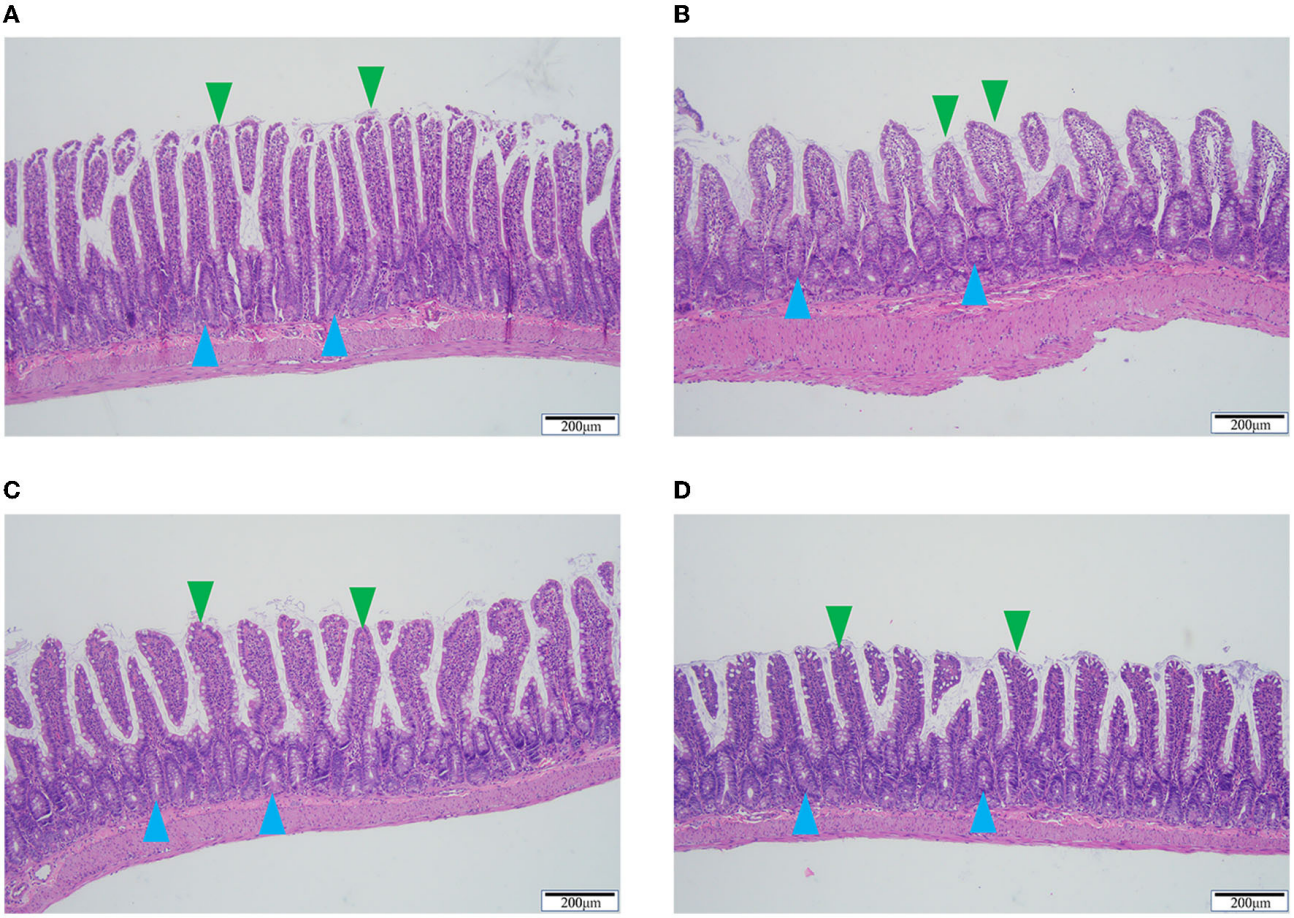
HE staining of ileum tissue. **(A)** Sham group. **(B)** Model group. **(C)** Qishen granule group. **(D)** Trimetazidine group. Blue arrowheads indicate the epithelium and green arrowheads indicate the ileum villi. Scale bar = 200 μm.

Compared to the sham group, the model group exhibited significantly lower epithelium and ileum villi heights. The ileum villi in the sham group were neatly ordered and tightly arranged, whereas in the model group they were rough, swollen, and irregularly arranged. QSG and TMZ improved the above pathological changes (Figure 4).

### 3.5. OTUs, Venn, and alpha microbial analysis

A total of 2,732,663 raw reads were obtained from intestine microbiota by 16S rRNA Illumina sequencing, with an average of 53,457 clean reads per sample after quality control and read assembly. The amount of sequencing data was sufficient.

**Figure 5:**
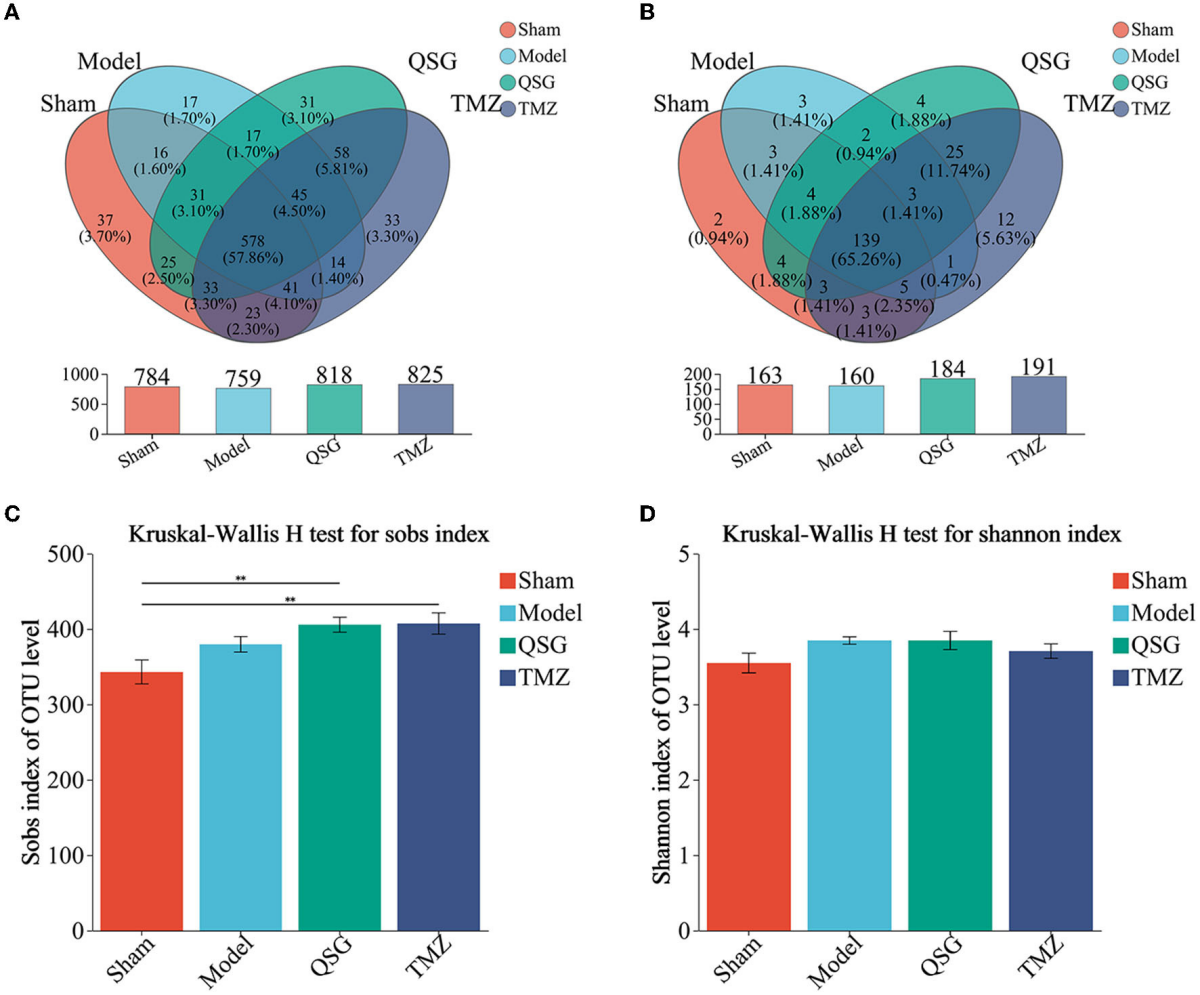
OTUs, Venn, and alpha microbial analysis. **(A)** Venn result of microbial analysis on the level of OTUs. **(B)** Venn result of microbial analysis on the level of genus. **(C)** Community richness. **(D)** Community diversity. *N* = 10 per group; QSG, Qishen granule; TMZ, trimetazidine.

In total, 999 OTUs were identified, with the Venn diagram showing that 578 (57.86%) ere shared among the four groups and 118 (11.8%) OTUs were unique to each group (Figure 5A). On the genus level, after matching and identification, 999 OTUs were mapped to a total of 213 genera, with 139 (65.26%) shared among the four groups and 21 (9.86%) unique to each group (Figure 5B).

Although there was no significant difference in community diversity (Shannon) among the four groups (Figure 5D), community richness (Sobs) was increased in the model, QSG, and TMZ groups compared to the sham group (Figure 5C). The rarefaction curve, constructed by community richness (Sobs), is shown in Supplementary Figure 3.

### 3.6. PLS-DA and percent of community abundance among the four groups

**Figure 6:**
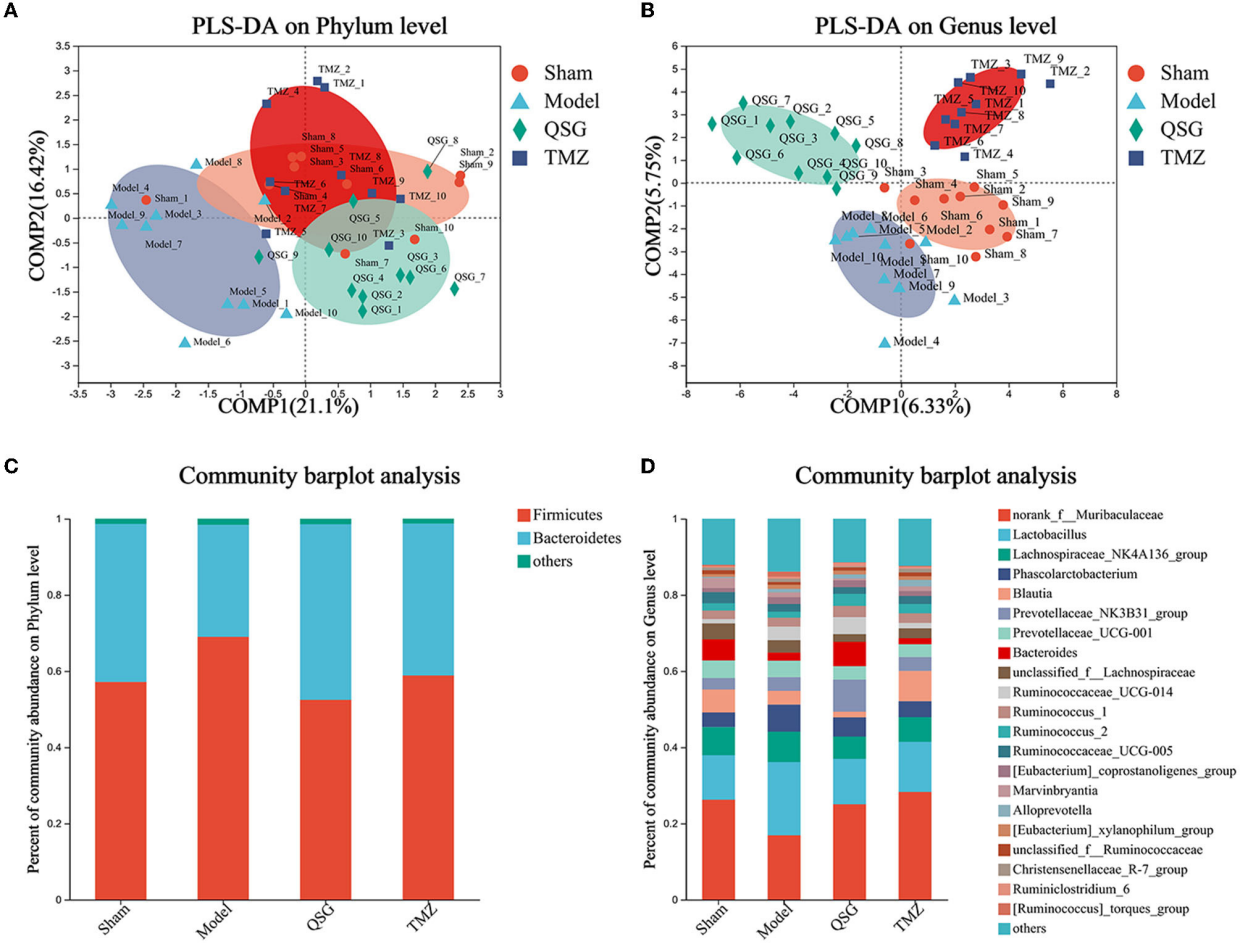
PLS-DA and percent of community abundance among the four groups. **(A)** PLS-DA analysis of the phylum level. **(B)** PLS-DA analysis of the genus level. **(C)** Percent of community abundance on the phylum level. **(D)** Percent of community abundance on the genus level.

PLS-DA analysis of the phylum level showed that the sham, QSG, and TMZ groups were clustered together, separated from the model group (Figure 6A), and that the main phyla were Firmicutes and Bacteroidetes (Figure 6C). PLS-DA analysis of the genus level showed that samples from the four groups were separated from others (Figure 6B), indicating that QSG and TMZ influenced the community composition. The main genus is shown in Figure 6D.

### 3.7. LEfSe and phenotype prediction among the four groups

**Figure 7:**
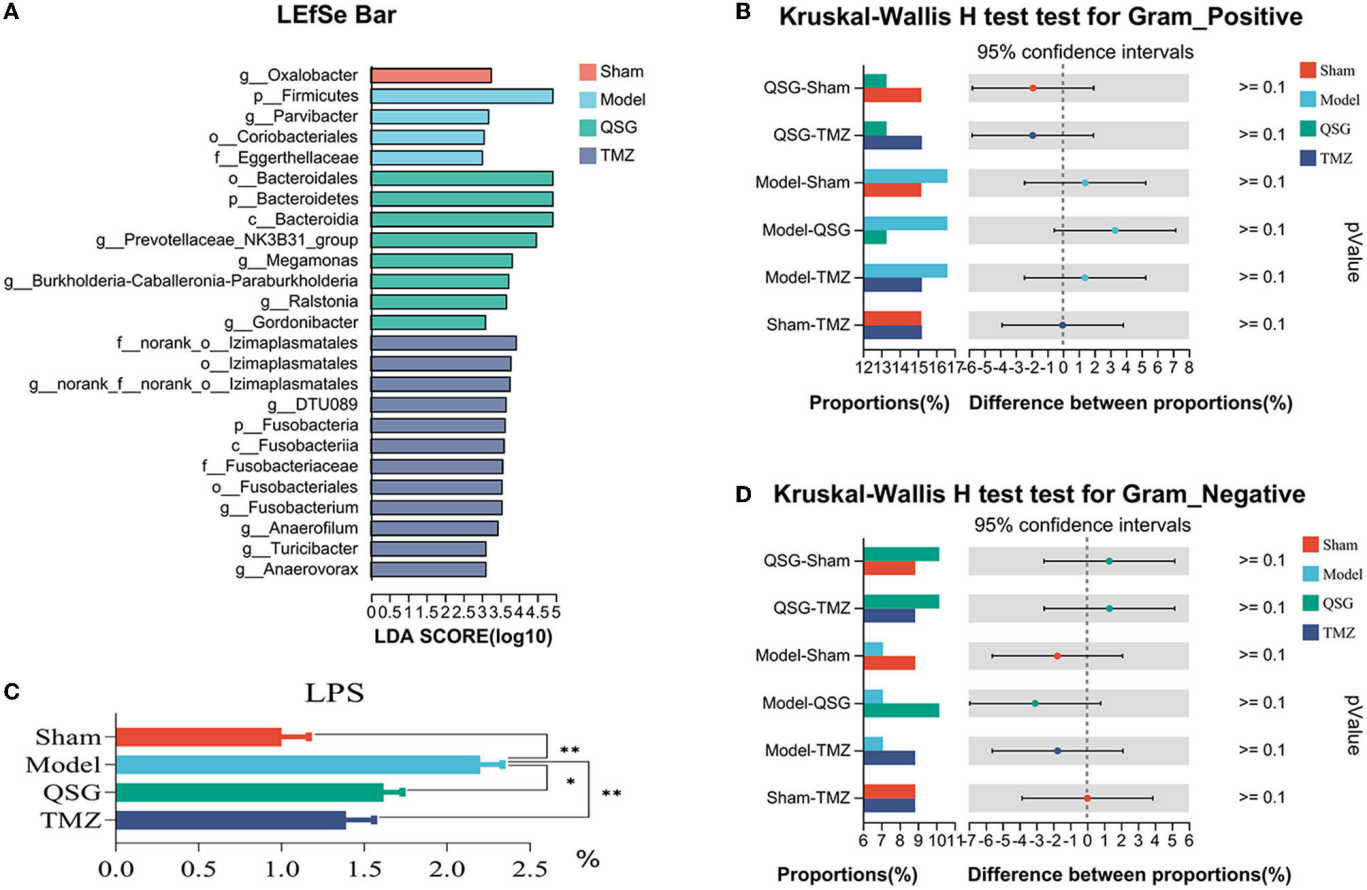
LEfSe and phenotype prediction among the four groups. **(A)** LEfSe analysis. **(C)** LPS detection. **(B, D)** Phenotype prediction. *P < 0.05, **P < 0.01; QSG, Qishen granule; TMZ, trimetazidine.

LEfSe analysis was used to screen microbes differentially among species. The bar chart indicated that 25 specific taxa were identified (threshold value of LDA = 3), with 1 in the sham group, 4 in the model group, 8 in the QSG group, and 12 in the TMZ group (Figure 7A). Furthermore, according to BugBase phenotype prediction, there was no significant difference in the composition of gram-negative or gram-positive bacteria among the four groups (Figures 7B, D). The determination of plasma LPS showed that QSG and TMZ significantly reduced LPS caused by HF (Figure 7C).

## 4. Discussion

Previous studies have shown that QSG is effective in treating HF (Wang B. et al., 2017; Chen et al., 2022; Li et al., 2022). However, the effect of QSG on intestinal microecology has not been confirmed. Therefore, this study aimed to elucidate the possible mechanism of QSG regulating HF in rats based on intestinal microecological changes. The findings can be summarized as follows: (1) QSG administration improved cardiac function, tightened cardiomyocytes alignment, decreased fibrous tissue and collagen deposition, and reduced inflammatory cell infiltration, basically consistent with previous study conclusions (Li et al., 2016). (2) The results of electron microscopic observation of mitochondria showed that QSG could arrange mitochondria neatly, reduce swelling, and improve the structural integrity of the crest. (3) Firmicutes were the dominant component in the model group, and QSG could significantly increase the abundance of Bacteroidetes and Prevotellaceae_NK3B31_group. (4) Furthermore, QSG significantly reduced plasma LPS, improved intestinal structure, and recovered barrier protection function in rats with HF.

The gastrointestinal barrier consists of mechanical, immune, chemical, and biological barriers. In a normal state, harmful intestinal substances, such as bacteria and their related endotoxins, are prevented from entering other host body tissues and the bloodstream through the intestinal mucosa (Sandek et al., 2007b; Lozupone et al., 2012). In recent years, an increasing number of studies have supported the role of the gut in the pathogenesis of HF, known as the “gut hypothesis of HF.” The hypothesis suggests that reduced cardiac output and increased systemic congestion may lead to ischemia and/or edema of the intestinal muscles with HF (Sandek et al., 2007a), characterized by hypoperfusion, ischemia, hypoxia of the intestinal mucosal, congestion, increased permeability, and reduced absorption of nutrients such as sugars, proteins, and fats. These factors can change the abundance and composition of gut microbiota, leading to increased bacterial translocation and circulating endotoxin, such as LPS (Andreas et al., 2014). Translocated microbiota and increased LPS exacerbate intestinal barrier function damage, stimulate an inflammatory response, and accelerate the pathological development of HF (Organ et al., 2016).

Bacteroidetes are absolutely dominant in gut microbiota, participating in the metabolism of various substances, fermenting carbohydrates, polysaccharides, steroids and bile acids, promoting the formation of intestinal mucosa vessels, preventing intestinal inflammation (Brown et al., 2019), maintaining intestinal physiological functions, and exerting a significant influence on hosts' health (Yu et al., 2015). Compared to healthy individuals, the abundance of Bacteroidetes (the genera Bacteroides and Prevotella) in patients with coronary heart disease was significantly decreased, and the ratio of Firmicutes/Bacteroidetes was increased (Emoto et al., 2017), which was associated with many potential cardiovascular diseases. Jie conducted a whole-genome study on fecal samples of 218 patients with coronary heart disease and 187 healthy individuals, showing that the abundance of Bacteroides and Prevotella was relatively reduced in the former group (Jie et al., 2017). Tan sequenced the gut microbiota of 36 patients with ischemic cardiomyopathy with different cardiac function levels and found that the proportion of Firmicutes was closely related to the occurrence of ischemic cardiomyopathy, with HF severity increasing alongside Firmicutes abundance (Tan, 2018). In Li's study on individuals over 60 years old, LEfSe analysis showed that Bacteroidetes were more abundant in the healthy group, while Firmicutes and Enterobacterium were more abundant in the HF group (Li, 2019). Romano identified eight intestinal bacteria belonging to Firmicutes, which significantly decomposed choline and produced trimethylamine (Romano et al., 2015), and then trimethylamine oxide (TMAO) promoted myocardial microangiopathy in non-ischemic HF patients. Elevated TMAO predicted adverse events in both non-ischemic and ischemic HF patients (Rhee et al., 2013). Yu found that the average abundance of Bacteroidetes was 66.23 ± 5.11% in the clinically healthy control group, 45.69 ± 4.63% in the coronary heart disease group, and 27.89 ± 2.39% in the coronary heart disease combined with the HF group (Yu et al., 2021).

Hypertension (Marques et al., 2017), hyperlipidemia (Suparna, 2015), and obesity (Rastmanesh, 2011) are common risk factors for HF, and the ratio of Firmicutes/Bacteroidetes increases with the worsening of adverse degree.

In this study, Firmicutes abundance was the dominant component in the model group, and QSG significantly increased the abundance of Bacteroidetes, which might be one of the targets of QSG for cardiac protection.

Purushe et al. stated that Prevotella was related to the biosynthesis of short-chain fatty acids (SCFAs), which could supply nutrients for intestinal epithelial cells and maintain the intestinal mucosal barrier and an acidic pH environment to prevent the invasion of related pathogenic microorganisms. A lack of SCFAs might reduce the protective effect of the intestinal mucosal barrier and lead to increased levels of enterotoxin (Purushe et al., 2010; Shen et al., 2017). Tang et al. (2018) found that dietary supplements provided with SCFAs within 24 h after myocardial infarction significantly reversed the high mortality and ventricular rupture rates caused by broad-spectrum antibiotics. Kovatcheva et al. found that sugar metabolism could be improved by supplementing with prebiotics containing Prevotella (Petia et al., 2015). In a study of patients with chronic renal failure, Xie et al. found that the abundance of Prevotellaceae belonging to Bacteroidetes decreased significantly (Xie, 2014). In the mouse model of ulcerative colitis induced by glucan sodium sulfate, disturbed gut microbiota might aggravate intestinal mucosal barrier damage by reducing the thickness of the intestinal mucus layer. Huangqin Decoction could significantly improve the abundance of Prevotellaceae and maintain the function of the intestinal mucosal barrier (Xu, 2018). According to Liu et al. (2018) and Smith et al. (2019), the abundance of Muribaculaceae and Prevotellaceae_NK3B31_group was closely related to the generation of SCFAs. Additionally, the reduced abundance of the Prevotellaceae_NK3B31_group was associated with inflammation (Wu, 2018). A study on Lingguizhugan Decoction showed that the herbs group could increase the abundance and diversity of gut microbiota in mice with HF, regulate the disorder, and increase the abundance of gut microbiota associated with SCFAs production, such as norank_f_Muribaculaceae and Prevotellaceae_NK3B31_group (Zhang et al., 2023). In this research, QSG significantly increased the abundance of Prevotellaceae_NK3B31_group.

Treg cells transcribe Forkhead box protein3 (Foxp3) and maintained human immune tolerance (Figueiredo and Schumacher, 2016), and the expression level was positively correlated with the relative abundance of Izimaplasmatales (Zhang, 2021). Izimaplasmatales were significantly underrepresented in the diabetes model (Niu et al., 2022) and obesity group (Zhang, 2021). Turicibacter was associated with the production of butyric acid (Zhong et al., 2015), and the colonization of *Turicibacter sanguinis* could reduce the overall triglyceride level and groin fat cell size of the host (Wu et al., 2020). In this study, the abundance of Izimaplasmatales and Turicibacter was the dominant composition in the TMZ group.

Pasini et al. (2016) compared 60 HF patients with healthy individuals and found that intestinal permeability in patients increased by 78.3%, and the number of patients with cardiac function grades III and IV (NYHA grade) was higher than those with grades I and II. Sandek's team showed that, compared to the healthy control group, intestinal arterial blood flow in patients with HF was reduced by 30% to 43%, and the decreased intestinal arterial blood flow was correlated with the severity of HF (Sandek et al., 2014). Intestinal transport function was reduced by 54% in patients with HF, and it was more notable in HF patients with edema (Sandek et al., 2012). These data imply that the assessment of intestinal barrier function may contribute to the understanding of the gut-directed treatment of HF. In this research, QSG could improve the intestinal morphology of rats with HF.

Gut microbiota participates in food digestion through two major metabolic pathways, including sugar and protein decomposition (Sekirov et al., 2010). Meanwhile, gut microbiota can affect the host in various ways. To associate with other organs, the gut microbiota needs to release signaling molecules, which in some cases are microbial physical compositions, such as LPS. LPS typically interacts with host cells' surfaces through pattern recognition receptors (PRRs) (Larsson et al., 2012). PRRs recognize pathogen-related molecular and can stimulate immune responses (Brown and Hazen, 2014).

The relationship between systemic inflammation and associated bacterial migration in HF has been observed and documented. Specifically, elevated levels of endotoxins, particularly LPS, in HF patients initiated signaling cascades that increased the production of cytokines, such as tumor necrosis factor-α (TNF-α) and aggravated HF (Niebauer et al., 1999; Sandek et al., 2007a). Moreover, LPS levels in the hepatic veins of HF patients were significantly higher than those in other circulatory sites, including the left ventricle and pulmonary artery, suggesting that HF exacerbation might result from excessive endotoxin influx from the gut into the bloodstream (Peschel et al., 2003), preliminarily confirming the link between gut microbiota and HF.

LPS induces pro-inflammatory damage by binding its lipid moiety, lipid A, to Toll-like receptor 4 (TLR4) (Poltorak et al., 1998). This binding leads to the recruitment of the adaptor protein myeloid differentiation primary response protein 88 (MyD88) to the cytoplasmic domain of TLR4, resulting in the activation of the transcription factor NF-κB (Liu et al., 2017) and the expression of NLRP3, GSDMD, and IL-1 in downstream inflammatory pathways, ultimately promoting HF development (Violi et al., 2023). Interestingly, lipid A exhibits structural variation. Lipid A in Bacteroides LPS is penta- or tetra-acylated, which reduces TLR4 responses (Vatanen et al., 2016; d'Hennezel et al., 2017; Wexler and Goodman, 2017).

In this research, QSG improved intestinal morphology and reduced serum LPS content in HF rats. In previous QSG studies (Chen et al., 2022; Li et al., 2022), GSG protected the heart by inhibiting the TLR4/NF-κB pathway in both rat and mouse models.

Astragalus camptoceras Bunge (Fabaceae) was one of the main herbs in QSG, and other drugs in it demonstrated therapeutic effects on the intestine and gut flora. On the basis of conventional anti-inflammatory drugs, Astragalus granules combined with Bifidobacterium quadruple viable tablets effectively treated ulcerative colitis patients, improving T lymphocyte subset levels, reducing inflammation, and inhibiting disease activity, with good safety (Zhu, 2022). Astragalus polysaccharide increased the abundance of Bifidobacteria and lactobacilli, while decreasing enterobacteria and enterococci in the stool of rats with ulcerative colitis, thereby improving intestinal flora imbalance (Liang et al., 2012).

## 5. Conclusion

The holistic view of traditional Chinese medicine posits that the host and its environment are inseparable, and although gut microbiota reside within the host's body, they also belong to the “external environmental conditions” (Gao, 2004). A dynamic balance between the host and gut microbiota is required for maintaining normal physiological functions.

HF pathogenesis is complex, involving various physiological reactions, metabolic pathways, and signaling pathways, and results from comprehensive functional disorders. Gut microbiota dysbiosis and its metabolites play crucial roles in the occurrence and development of HF. Interventions targeting gut microbiota dysbiosis, improving intestinal barrier function and permeability, and reducing endotoxin absorption and inflammation may alleviate myocardial damage, suggesting a novel approach to HF treatment. The data presented here demonstrate that QSG improves cardiac function by regulating intestinal microecology in HF rats, highlighting promising therapeutic targets for HF.

However, it is important to recognize that the disease mechanisms caused by gut microbiota dysbiosis have not been fully elucidated and targeted interventions for gut microbiota in treating cardiovascular diseases, such as HF, have not been widely implemented in clinical practice. Therefore, the causal relationship and more detailed mechanisms between gut microbiota, their metabolites, and HF require further investigation.

## Data availability statement

The datasets presented in this study can be found in online repositories. The names of the repository/repositories and accession number(s) can be found in the article/Supplementary material.

## Ethics statement

The animal study was reviewed and approved by Beijing University of Chinese Medicine.

## Author contributions

KG, RY, and CW conceived this research. KG, XY, FL, YH, JL, and SL contributed to the process of experiment, sample collection, and data analysis. The first version of the article was written by KG and revised by XY. FL and LL performed the final review and contributed to the project administration. All authors contributed to the article and approved the submitted version.
