# Supplementary material for: Qishen granules regulate intestinal microecology to improve cardiac function in rats with heart failure
Source: Front Microbiol. 2023 Jun 15;14:1202768. doi: 10.3389/fmicb.2023.1202768 (PMC10307979; doi:10.3389/fmicb.2023.1202768)
Supplement: Supplementary file 1 [file Data_Sheet_1.pdf]

Supplementary material

2 Materials and methods

2. 1 DNA extraction and PCR amplification

Microbial community genomic DNA was extracted from rats feces samples using the E.Z.N.A.® soil DNA Kit (Omega Bio-tek, Norcross, GA, U.S.) according to manufacturer's instructions. The DNA extract was checked on 1% agarose gel, and DNA concentration and purity were determined with NanoDrop 2000 UV-vis spectrophotometer (Thermo Scientific, Wilmington, USA). The hypervariable region V3-V4 of the bacterial 16S rRNA gene were amplified with primer pairs 338F (5'-ACTCCTACGGGAGGCAGCAG-3') and 806R(5'-GGACTACHVGGGTWTCTAAT-3') by an ABI GeneAmp® 9700 PCR thermocycler (ABI, CA, USA). The PCR amplification of 16S rRNA gene was performed as follows: initial denaturation at 95 °C for 3 min, followed by 30 cycles of denaturing at 95 °C for 30 s, annealing at 55 °C for 30 s and extension at 72 °Cfor 45 s, and single extension at 72 °C for 10 min, and end at 10°C. The PCR mixtures contain 5 × *TransStart* FastPfu buffer 4 μL, 2.5 mM dNTPs 2 μL , forward primer (5 μM) 0.8 μL, reverse primer (5 μM) 0.8 μL, *TransStart* FastPfu DNA Polymerase 0.4 μL, template DNA 10 ng, and finally ddH$_2$O up to 20 μL. PCR reactions were performed in triplicate. The PCR product was extracted from 2% agarose gel and purified using the AxyPrep DNA Gel Extraction Kit (Axygen Biosciences, Union City, CA, USA) according to manufacturer's instructions and quantified using Quantus™ Fluorometer (Promega, USA).

2.2 Illumina MiSeq sequencing

Purified amplicons were pooled in equimolar and paired-end sequenced (2 ×300) on an Illumina MiSeq platform (Illumina, San Diego,USA) according to the standard protocols by Majorbio Bio-Pharm Technology Co. Ltd. (Shanghai, China).

2.3 Processing of sequencing data

The raw 16S rRNA gene sequencing reads were demultiplexed, quality-filtered by Trimmomatic and merged by FLASH with the following criteria: (i) the 300 bp reads were truncated at any site receiving an average quality score of <20 over a 50 bp sliding window, and the truncated reads shorter than 50 bp were discarded, reads containing ambiguous characters were also discarded; (ii) only overlapping sequences longer than 10 bp were assembled according to their overlapped sequence. The maximum mismatch ratio of overlap region is 0.2. Reads that could not be assembled were discarded; (iii) Samples were distinguished according to the barcode and primers, and the sequence direction was adjusted, exact barcode matching, 2 nucleotide mismatch in primer matching.

Operational taxonomic units (OTUs) with 97% similarity cutoff (Liu et al. 2017) were clustered using UPARSE (version 7.1, http://drive5.com/uparse/), and chimeric sequences were identified and removed. The taxonomy of each OTU representative sequence was analyzed by RDP Classifier (http://rdp.cme.msu.edu/) against the 16S rRNA database (Silva SSU132) using confidence threshold of 0.7.

3 Experimental Results

3.1 Evaluation of disease model by Electrocardiography

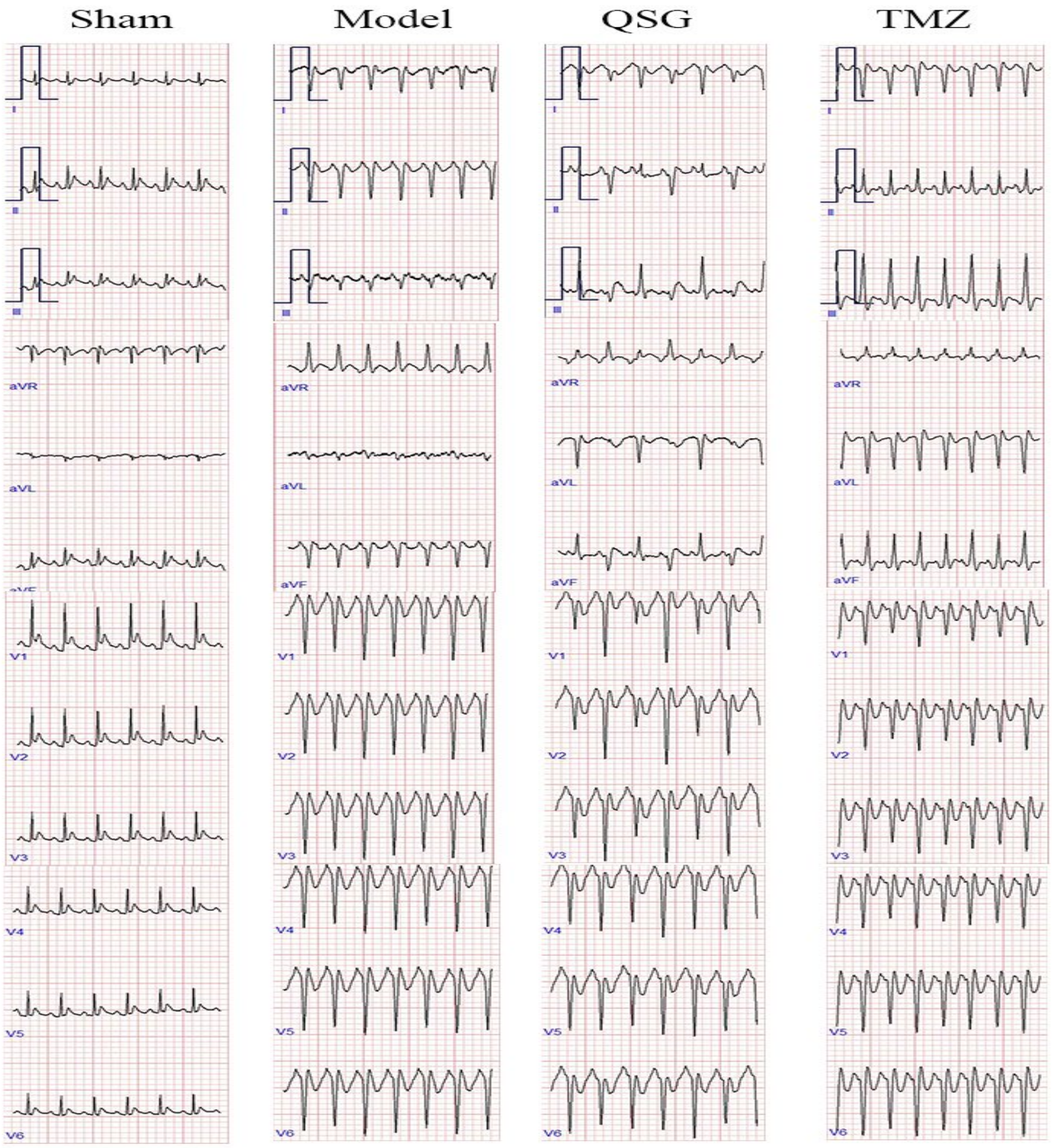

Supplementary Figure 1. 12-lead electrocardiogram 3 days after operation

Notes: QSG, Qishen granule; TMZ, Trimetazidine

In the Supplementary Figure 1, the electrocardiogram of the sham was roughly normal, and there were pathological Q wave at I, aVL and V2-6 leads in the model, QSG and TMZ groups. It indicated that the operation was successful.

3.2 Weight changes of four groups

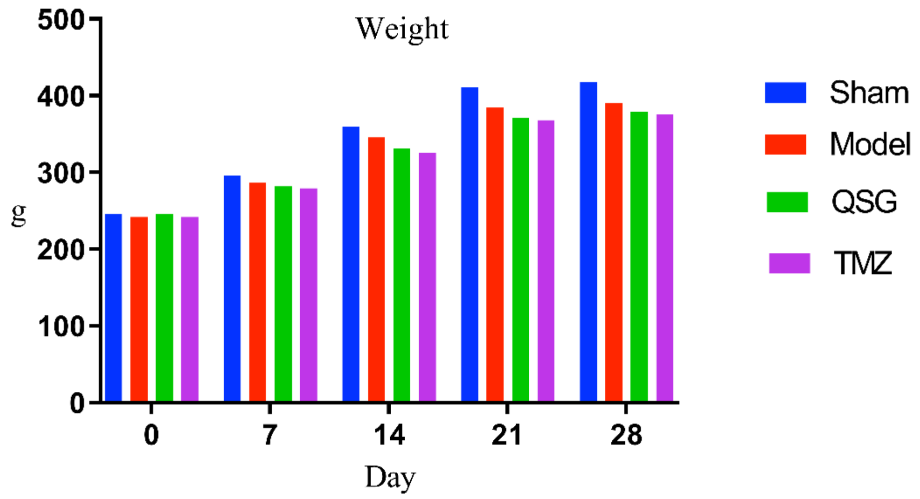

Supplementary Figure 2. Weight changes of four groups

Notes：compared with the model group, *P<0.05; QSG, Qishen granule; TMZ, Trimetazidine

In the Supplementary Figure 2, there was no significant difference in body weight among the four groups at different time points.

3.3 Rarefaction curve

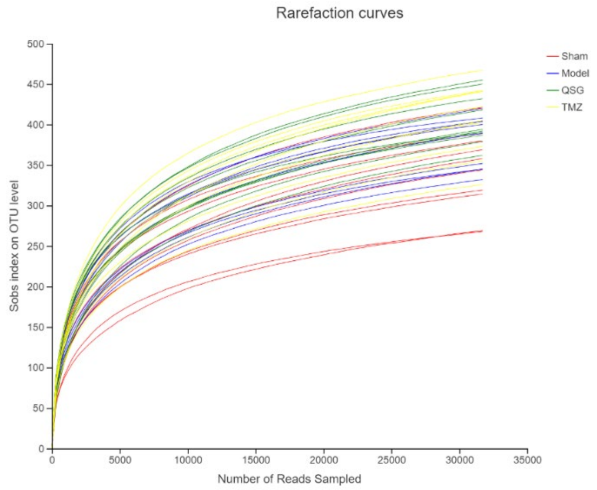

Supplementary Figure 3. Rarefaction curve

Notes：QSG, Qishen granule; TMZ, Trimetazidine

In this research, the rarefaction cure was constructed by the Alpha diversity index sobs, and according to the Supplementary Figure 3, the curves of 4 group of samples tend to be horizontal, indicating that the size of sequencing data was reasonable.
